# The Present Status of Antiseptic Surgery*Read at the regular meeting, October 16, 1882.

**Published:** 1882-10

**Authors:** Roswell Park

**Affiliations:** Lecturer on Surgery, Rush Medical College, Surgeon to the Michael Reese Hospital, etc.; 1558 Wabash Avenue


CHICAGO MEDICAL

JOURNAL & EXAMINER.

Vol. XLV.—OCTOBER, 1882.—No. 4.

### Original Communications.

Article I.

### The Present Status of Antiseptic Surgery. A Report made by Request of the Chicago Medical Society,* by Roswell Park, A.M., M.D., Lecturer on Surgery, Rush Medical College, Surgeon to the Michael Reese Hospital, etc.

In making this report, I prefer to omit all theoretical discussions concerning the bearings of the germ theory, and the influence of germs in producing fermentation or decomposition, often carried to putrefaction. The acceptance of this so-called theory—which is in reality no longer a theory, but an incontestably proved fact—is so necessary for the proper practice of antiseptic measures, that he who cannot, so to speak, take the oath of allegiance to it, is committing a gross inconsistency in adopting in his work any of the proper antiseptic details. Moreover, this part of the subject will in due time be much more ably handled by another member of this society.

* Read at the regular meeting, October 16, 1882.

Accepting, then, this theory as the firm basis of fact to which we cling, it remains for us to study how we can best strengthen our position, and better prepare for a conflict in which knowledge is arrayed against overwhelming, and even infinite numbers.

To what extent do statistics help us? They serve to strengthen our convictions, but I imagine that few men began to use the Lister dressings impelled by a mere study of statistics. In fact, up to a very recent time, very few have appeared from the fountain head. Until Bryant, Savory and others provoked Mr. Lister into giving his results in figures and tables, we had to depend largely on those furnished from the German clinics. But very recently a work by Mr. Cheyne * has appeared, which contains such a mass of statistics on the subject that from its study even the most doubting Thomas amongst us must needs be convinced, and which should be regarded as having as much weight and importance as if Mr. Lister himself had written it, coming as it does from the pen of his trusted assistant and co-laborer. In this book the tables are so admirably arranged, and the details are so readable, that to it I would unhesitatingly refer him who desires to investigate the numerical features of the case. The work is both later and larger than that of MacCormack, and must therefore replace it. Besides these, we have the recent utterances of Volkmann,† Esmarch, Nussbaum,‡ Neudörfer § and others, which are of very great importance, and classic in their way.

Before considering applied details, it may be of advantage if we refer to the appearance and reactions of some of those minute organisms whose uninvited and unperceived presence often renders our best efforts futile. Can their presence be in any way detected, and can we distinguish between the innocent and the guilty?

It may be accepted as a general rule that their shape and size give reasonable indications as to their character. The nearer they approach the spherical or dumb-bell shape, the less injury

---

* *Antiseptic Surgery. Its Principles, Practice, History and Results.* By Watson Cheyne, M.B., F.R.C.S., etc. London: Smith, Elder & Co. 1882, pp. 600.

† Sammlung Klinischer Vorträge, No. 96.

‡ Leitfaden zur Antiseptischen Wundbehaudlung.

§ Die chirurgischer Behaudlung der Wunden.

they do; while the more slender and rod-shaped they are, the more danger they betoken. The case reported at the last meeting of the society shows that spherical micro-organisms may exist in the blood of patients who give no external evidence of disease, while I think no case has ever been reported where the bacterium termo has been found in the blood of healthy individuals. Careful examination will reveal the former, too, in many wound secretions which have a sour smell, though the reparative process is not apparently delayed thereby, nor the farther decomposition or blood-poisoning take place. Their reaction to staining fluids and methods of cell division also denote wide differences between the different forms. I can testify unequivocally to the existence of living spherical organisms in the freshly drawn blood of the patient whose case of tetanus was reported to you two weeks ago, having been shown them since then by Prof. Curtis, while the boy certainly appeared in excellent health. Regarding these, then, as comparatively or quite innocuous, let us devote a moment to the rod-shaped bacteria. Are they ever present in the healthy living body, and must they inevitably lead to septic decomposition when present? Beside the above statements that they have not been so found, let me direct your attention to certain experiments made by Chauveau, and give briefly his "*experience du bistournage.*"

*Bistournage* is performed by subcutaneously rupturing the spermatic cord by torsion. The testicle thus freed lies separated from nearly all its sources of nutrition, but is protected by its tunics from access of air, which means from access of germs. Its adhesions to the tunica vaginalis are not sufficient to nourish it, and consequently it atrophies. If the blood contain putrefactive germs, then this testicle ought to break down. This it does not do; no germs reaching it either from the blood or the air; it simply disappears by absorption. But if the germs come from without the organism, the fact can be proved as Chauveau proved it. He injected septic organisms into the vascular system of rams. After the fever, when not fatal, had subsided, he performed the *bistournage*, and putrefaction of the testicle ensued. But this was not enough. Lest some might object that the bacteria in the injected fluid were not the active agents, he carefully filterd the liquid to remove them, and then repeated the experiment, but never got the same result. To make assurance surer, and to prove that it was not the infective fever which caused the putrefaction of the organ, he varied the experiment in this way: He injected two rams with the same fluid and same dose, and then made the *bistournage* on one, with the result that putrefaction followed only in this one case. Furthermore, when he practiced the *bistournage* on the left testicle before, and on the right after injection, it was the right only which putrefied.*

Here was a most ingenious proof that the admission of such germs into an organ determines its destruction. Should any one ask how, then, it happens that, when these germs can effect destruction of parts, they do not destroy life, the answer is at hand; first, we know that life frequently is lost in this way and from this cause; and second, we know how vigorously we have to combat these organisms when we succeed in saving our patient.

It will be profitable next to consider how it happens that after injection of irritating substances, such as *e. g.*, ammonia, we find septic micro-organisms in the pus thus formed. Without stopping in this place to go over the experimental ground, therefor, we are forced to believe that such germs have more or less access to the blood, via the lungs and alimentary canal, on account of their minuteness, but that the blood and tissues in a healthy state offer so poor a field for their development that they die from lack of favorable surroundings.

(No reference is made here to the special infectious diseases, each of which is supposed to have its own bacillus, though the fact that the infection does not always follow exposure can be thus best explained.) But in condition of lowered vitality, depraved nutrition, or in the presence of other diseases, the blood and tissues may become so insensibly altered that it assists, instead of preventing their growth. In actual practice we see sepsis and erysipelas occur much more frequently in the exhausted, the debilitated and the aged than in the young and healthy. No other conclusion than this satisfactorily accounts for known facts.

---

* Quoted from Jeannel's "De l'Infection Purulente." (Cheyne.)

Another point: The sphero-bacteria seem to be more or less inimical to the more poisonous rod-shaped bacteria; the former very rapidly exhaust the nutritive fluid in cultivating solutions and starve out the latter. It may be a most fortunate thing, then, when the former are present in the blood and wound secretions, for they may serve thus to make things unpleasant for the latter, and either prevent their appearance or hasten their disappearance.

In surgical injuries, micro-organisms may enter the lists through two different paths; through the blood, or by the wound. We take precautions against the first possibility in three ways. (1). We endeavor, as far as possible, to operate upon patients only when they are in good health. (2). When they are reduced or aged, we try to put them through a "building up process," by which we may restore to blood and tissues that unknown element vitality, that imparts this resistant power. (3). And when emergency compels us to operate upon those in unfavorable condition we try to impart this property in the speediest possible manner by administration of those remedies which are both stimulant and antizymotic, such as alcohol, quinia and the like.

As against infection of the wound surface, we have the chain of precautions known as "Listerism," a system which, when scrupulously carried out, makes the entrance or lodgment of any living germ very improbable, perhaps impossible. This system has won its way over incredulity and ridicule, and has left an impress which shall prove an historical monument, no matter what change it may undergo.

Following Mr. Lister and his disciple, Mr. Cheyne, we ought to distinguish between aseptic and antiseptic surgery. The former includes a series of measures calculated to absolutely exclude every living germ from lodgment on the wound surface or in the dressings. To this end there is probably but one known method, and that is the strictest form of Listerism. It is, of course, impossible to say to exactly what extent any of his details could be omitted, or whether any others could be substituted for them; but those who aspire to make the course of a wound perfectly aseptic had best adhere strictly to those directions laid down by their author. It would take a long course of experimenting, which might be trifling with human life, before we could deviate from his plan. Besides, no one could go over the question more carefully than he has done. These details are laid down in so many perfectly accessible places that it would be superfluous for me to recount them here. Let us remember that their strict and conscientious observance constitutes our only positively aseptic surgery, and, turning our attention to the other part of the subject, consider how we can best render our operative procedures and our subsequent dressings, properly speaking, antiseptic.

Here a much wider field is opened up to us. Moreover, it makes comparatively small difference whether we positively exclude germs or cripple and destroy them when present; whereas, the latter course is the easier, and the good we can accomplish by devising simple and easily applied dressings which may be brought into extensive common use, and which shall be genuinely antiseptic, will greatly overbalance any harm that may happen in exceptional cases by omitting to resort to the complicated and expensive Lister method. In other words, we shall by the one practice save many more lives than we shall lose by not adopting the other.

When such a statement can be made as was heard at a recent congress of German surgeons, that little or nothing was now left of the original Lister system among those who were its most firm adherents, it behooves us to find out whether this is really the case, as well as to inquire why these features have been one after another dropped or changed. I propose, therefore, to speak of each of them separately, and then of their various substitutes.

And first of the spray. The cry of "Fort mit dem spray" was raised some little time ago, and we are now told that Mr. Lister himself considers it the least essential part of his system. Several causes have conspired toward its abandonment. In the first place, numerous cases of carbolic acid poisoning were reported as owing to its use. Then grave doubts arose as to its being so effective as at first supposed. Besides this, it was not only unpleasant for the operator, but often a positive disadvantage to him. As a result of all this, the atomizer is seldom seen in the German clinics. It is chiefly used during operations where serous membranes are exposed. In the service of Billroth, Albert, Gussenbauer, Czerny, Luecke, Nussbaum and many others, it is very seldom used during any but those just mentioned, and by no means always during those. A very frequent practice with those surgeons is to keep very large atomizers playing in the operating room for an hour or so beforehand, especially when laparatomy is to be made; but when the patient appears the machine is removed. Some of the objections to the spray may be overcome by substituting some other antiseptic for the phenol. Thymol has proved as worthy as its former reputation; salicylic acid is better; acetate of alumina has not yet had sufficient trial; oil of eucalyptus bids fair to be a success. In Paris, Pean is using peroxide of hydrogen, but the substance is very unstable, and he nas no better results than many who use no spray. Altogether, the present tendency is to make the use of the spray the exception, rather than the rule.

The protective was an essential feature two years ago. Now it is much less often used. It was supposed to fulfill two indications; to protect the raw or tender surface beneath it fiom the rather irritating carbolized gauze, and by the presence of lead oxide in its coating to indicate when decomposition had taken place. We now find that it is not necessary to apply gauze directly to the skin, for a layer of borated cotton is better than both. Then the drainage tubes used are often made of black rubber, which contains more or less free sulphur, and this, meeting with the lead oxide, gives a false indication of decomposition. The nose of a healthy individual is a sufficiently accurate detector of putrefactive changes. The protective serves this useful purpose, however, it keeps the cotton fibers from adhering to the edges of the wound, to hairs, or ends of sutures, and thus makes change of dressings much less unpleasant to the patient; for this purpose it should be often used.

No noteworthy change has been made in the use of drainage tubes. But they should be made of red rubber instead of black, for the reason above stated; their ends should be cut off even with the surface, that they may not be occluded by pressure, and should be held in place with a single suture through the edge of the wound.

The gauze perhaps holds its own. It is used just the same as ever. But it is in no way superior to well made boro-salicylic cotton, while the latter is much the softer. Then, too, we have finely picked and prepared oakum, tow and jute, all of which are admirable, and cheaper than fine cotton. They may, therefore, be laid over the first layer of cotton.

Cheaper substitutes for Macintosh are now made in abundance; we have very thin rubber cloth, gutta-percha paper, waxed paper, and that prepared with paraffine; and all, save the latter, pliable and supple.

Last of all, for the outside bandages, the Germans are now using a very fine, thin sort of muslin goods, which are soaked in carbolized water just before using, and can then be snugly and easily applied. An elastic rubber bandage may occasionally be used to advantage.

All these materials we may regard as, in a measure, duplicates of the materials employed by Lister. Now, as we gradually depart from his original methods, numerous other ways and means of attaining nearly or fully his results are before us. Some of these are well worth our attention. A very important question for us to ask ourselves is this: If the spray is of doubtful utility, do we need any substitute? Probably we can accomplish in most, if not in all cases, all that is necessary by practising irrigation with carbolized water, This may be done occasionally during the operation, and should always be done just before applying the dressings. If the hands, instruments and sponges be perfectly clean and then carbolized, as well as the water in which the latter are cleansed, we need feel little fear for the result. And if the water used for irrigation be quite hot, and allowed to play in a fine stream upon the part during the operation, we shall have an efficient antiseptic and styptic both.

Of the various antiseptics the following deserve consideration to-night: thymol, potassium permanganate, aluminum acetate, zinc chloride, resorcin, salicylic acid, alcohol, glycerine, eucalyptol, boracic acid, napthalin and iodoform.

*Thymol* was first brought to notice for this purpose by Volkmann and Ranke, of Halle.* At first it promised well, but it has not stood the tests and is now seldom used. Once in a while it is used in the spray during laparotomy, but the solution ought to be as strong as 1 to 250 instead of 1 to 2,000, as at one time recommended.

*Potassium permanganate*, in solution, serves very well for irrigation and washing out purulent cavities ; but it is seldom or never employed in the spray. Its solution is excellent, too, for cleansing sponges, or for keeping them.

*Aluminum acetate* was introduced by Maas, who uses 2½ per cent. solution in spray, and soaks his compresses and dressings in the same. He claims that his cases follow a strictly antiseptic course, while the salt certainly is free from irritating qualities. It is deserving of further trial.

Solution of *zinc chloride* was long ago recommended by Lister for cleansing and purifying of wounds and sinuses that had become more or less foul. Recently, Kocher† has published his experience with the use of weak solutions in the clinic at Bern. He often uses them as weak as one-fifth of one per cent., and saturates his dressings with them, thus making it another substitute for carbolic acid. If one may judge from his published statistics, his results are most satisfactory.

*Resorcin* is a new candidate for favor. The writer has had no personal experience with it, and has hardly seen enough reports of its use to be able to speak intelligently thereof. Like many other new drugs, if all that is said of it be true, it must be of value; but manufacturers' and dealers' statements are not always reliable.

*Salicylic acid* has the advantage over carbolic acid that neither in strong solution nor in powder has it odor or caustic effect; its disadvantage is its slight solubility in water. It was brought into surgical use by Thiersch,‡ of Leipzig, and has been used a great deal. In the spray it is non-irritating to raw surfaces and serous membranes, and is not infrequently employed. In

* Volkmann's, Samml., Klin. Vort., No. 128.

† Volkmann's, Samml., Klin. Vort., No. 203–4, 1881.

‡ Volkmann's, Samml., Klin. Vort., No. 84–5.

Langenbeck's clinic a reservoir of it was always at hand for this purpose. Volkmann has showed that in those cases which I have already referred to where the discharges have a sour odor, and sphæro-organisms are found under the microscope, the use of this acid checks all mischief and odor. Lister makes a cream or ointment of this acid to apply around the edge of other dressings where irritation has been set up. It may be used to advantage in solution for irrigation where carbolic acid is irritating or obnoxious, and dusted dry on ulcerated surfaces, where it dries into the crust and forms a perfect protection from the air.

*Alcohol* is a most efficient antiseptic; the name of the individual who introduced it having been lost in the dawn of history. As a stimulant injection, more or less dilute, its use is familiar to all. It is desirable here to lay stress on the fact which Hack* has demonstrated, that granulations treated by alcohol absorb very little, if at all, while those treated by carbolic acid absorb easily. The practical importance of this fact is very great.

*Glycerine* has, in the writer's opinion, been too much neglected. Introduced for this purpose by Demarquay, in 1855, it was first tried in hospital gangrene, and then in all wounds. He esteemed it and praised it most highly.† I have used it considerably in preserving anatomical specimens and in embalming, and I have usually found that the more severe the test the more satisfactory the result. I had for some time been using carbolized glycerine as a substitute for carbolized oil, it being much more cleanly, and miscible with the water used in washing or irrigating, when I met with articles on the subject by Griffin‡ and others. They advocated its use in major operations, such as severe amputations, etc., claiming that they thus got union by first intention just as when they used the Lister dressing. It has this great advantage that it does not evaporate, and that when mixed with a due proportion of carbolic acid, or alcohol,

---

* Ueber das Resorptionsvermögen granulirinder Flächen." Leipzig, 1879.

† Gazette des Hopitaux. Octobre, 1855.

‡ Lancet, June 18, 1881, p 985.

or iodoform, dressings saturated with it must be either impermeable to the air, or must act as antiseptic filters.

I now thus use it a good deal as a dressing for lesser amputations, lacerated or contused wounds, plastic operations, and the like. I saturate soft canton flannel, or surgeon's lint, or absorbent cotton, with it, apply two to six layers, and then put some gutta-percha paper over it, or not, as the case may seem to require, and over all this the usual bandages. With this dressing I have had no bad result, but as complete union by first intention as I ever saw. I can't help venturing the statement, therefore, that the more anatomists and surgeons use glycerin the better they will like it.

Preparations from the eucalyptus, especially the oil, are gradually working themselves into favor. Schulz, of Bonn, urges their adoption.* Baucholtz and Siegen have proved their antiseptic properties. They also hinder the emigration of white blood corpuscles, and can thereby influence suppuration.

Schulz directs that for the spray the glass reservoir of the atomizer should be filled with an alcoholic solution of the oil which the steam could take up and emulsify. Its odor and expense serve to tell against it. A ten per cent. solution of the oil may be used, as I have just recommended, with glycerine. It may also be used in the preparation of gauze. Lister's own experiments with it have proved, according to Cheyne,† very encouraging. It may prove a substitute for carbolic acid under all circumstances. Its non-poisonous and anti-malarial properties should be greatly in its favor.

*Boracic acid* is too sparingly soluble to be of much service save where it may be applied in dry powder, but in such cases it hardly has a superior. Its crystals are difficult to pulverize, but this difficulty may be overcome by trituration with a few drops of alcohol. It may then be dusted from a pepper-box or blown through a blower. Cavities that are to heal by granulation may be filled with it. Thus used after excisions, sequestrotomies, removal of projectiles, etc., it offers the safest means of preventing sepsis. For such purposes its only rival is iodoform, and it has

---

* Centralblatt für Chirurgie, Jan. 24, 1880.

† Loc. cit. p. 141.

this great advantage, that it is odorless, and that boracic acid poisoning is almost unheard of.* Lint and cotton may be steeped in hot saturated solution, after cooling and drying the fine crystals are deposited in the meshes of the fibre. Still better is the result if salicylic acid be added. Absorbent cotton thus prepared is equal to the best Lister gauze. Boracic acid is not yet used nearly as much as it ought to be. It has very similar advantages to iodoform without its odor. It certainly stimulates healthy granulations. For my own part, I like it better and better as I use it more and more.

*Napthalin*, I imagine, has not yet been tried this side of the ocean. Discovered by Garden in 1820, recently introduced by Luecke, of Strasburg, and described by Fisher,† it is a very cheap and efficient "antibacteriticum." It is a "coal-tar product," and has the chemical formula, C.10, H.8. Like boracic acid, it is with difficulty reduced to fine powder, but may be used in just the same way and with the same results. Its advantages over everything yet mentioned in this paper is its cheapness. It is a white crystalline body, melting at 79° C., insoluble in water, but easily soluble in four parts of ether. Gauze may be impregnated with a mixture of this solution, with three parts of alcohol. This gauze will cost one-fifteenth as much as iodoform gauze. Napthalin is easily miscible with unguents and may thus serve in many parasitic and other skin diseases. (Kleinhaus, Simon.) I saw Luecke using gauze which had been treated with the drug reduced to as fine powder as possible, and simply rubbed into its meshes, just as plaster of Paris bandages are made. With this he was dressing all his cases. Fischer relates cases where the use of napthalin locally has suddenly checked erysipelatous processes. It can be used in any and all ways. I shall soon begin using it myself, and hope to see it generally tried.

Lastly, we have *iodoform*. So much has been of late written‡ about this substance that I need not devote much time to it;

*In the St. Petersburg Vratsch, No. 31, 1881, two cases are reported.

† Berlin. Klin, Wochenschr., Nov. 28, 1881; and Centralblatt für Chirurgie, Beilage, No. 29, 1882.

‡ Langenbeck's Archiv. XXVII, p. 196 Wiener Med. Woch., Nos. 13 and 23, 1881. Weiner Klinik, Jan. 1882. Volkmann's Samml., Klin. Vort. No. 211. Vide also Annals of Anat. and Surg., March and July, 1882.

German current literature contains very frequent allusions to it. When we realize that the iodoform dressing is used most extensively all over Germany we must feel that its merits are very great. As an antiseptic it is regarded equal to carbolic acid, and it is perhaps less liable to cause constitutional disturbance. Still, symptoms of poisoning are now and then observed. Iodoform is absorbed in small quantity, and must accordingly be eliminated in some way. This elimination takes place through the kidneys. Moosetig-Moorhof, who introduced it, claims that this free elimination is prevented by the carbolic acid used during operations, since it irritates these emunctories; he, therefore, uses only clear water for irrigation, instruments and sponges. Without being prepared to pass judgment on his views. I can only say that they do not seem to be shared by those surgeons who, besides himself, most use it.

The introduction of iodoform has certainly advanced the possibilities of surgery. The great danger formerly after such operations, as extirpation of the larynx, thyroid or rectum, excision of the tongue, and the like, was from purulent infection, because by the very nature of the cases a strictly antiseptic dressing was impossible. Now, by the use of this same drug they are made practicable and even safe. I have seen Billroth stuff a tent of iodoform gauze into the peritoneal cavity after extirpation of the uterus per vaginam, and, leaving it there, tampon the vagina with the same,—the patient recovering without a drawback. After operations about the mouth, the wound is stuffed with this gauze, which is changed only after two or three days. After laparotomy, iodoform is sprinkled into the abdominal cavity without hesitation. In a similar manner it is employed after plastic and osteoplastic operations. The various opportunities for its use in syphilitic and skin diseases can only be hinted at here.

In spite of these advantages, it has some bitter enemies, but it is steadily gaining ground. In the writer's humble opinion it is *the* antiseptic, for surgical purposes, of the immediate future. It will find its largest field, perhaps, in cases of tuberculous joints, sinuses and ulcers. Indeed, its friends claim for it positively and specifically anti-tuberculous effects. This matter should be regarded as still *sub judice.*

The only serious charge which can be brought against it is that under its use erysipelas happens more frequently than when the Lister gauze is used. Reports indicate that this is rarely the case; and yet it happens so very seldom that iodoform can still hold its own against competitors.

Iodoform gauze may be prepared in two ways. The powdered drug may be sprinkled over the proper material and rubbed in. Or, better, 60 grammes of resin are dissolved in 1200 gr. of alcohol, and 50 gr. of glycerine added; into this fluid 6 meters of gauze are placed, and then wrung out; when half dried it is dusted with 50 gr. of powdered iodoform.

Concerning the quantity of drug that it may be considered safe to use, those who most use it have set the limit of absolute safety at 10 grammes, yet much more may be tolerated, though seldom required. Indeed, Gussenbauer* poured 240 grammes (nearly eight ounces) into the cavity left after resection of the ankle, and no poisoning resulted.

Its penetrating, tell-tale odor can be partially disguised by balsam of Peru, eucalyptus oil, and various other liquids and solids.

Space fails to more than mention peat or black earth, corrosive sublimate*, terebene, sanitas, the various balsamic preparations, chloral, oil of mustard, and other aromatics, chinolin, iodine and many other drugs and chemicals that have at different times been recommended. They probably all have certain good points, but it is, to say the least, doubtful whether any of them can be compared to those which I have spoken of at some length.

Neither is this the appropriate place to discuss the relative merits of silk, cat gut, chromic gut, silkworm gut, nor ox-aorta ligatures, nor of those of deer or kangaroo tendons, or the like. No particularly new method of rendering any of them antiseptic has been lately presented, and it is with precautions against sepsis alone that this paper deals. The same remark applied to bone and other drainage tubes.

---

* Pers nal communication.

† These two recommended respectively by Neuber and Kümmel, Centralblatt für Chirurugie, Beilage No. 29, 1882.

I am fully aware that I have really only introduced the subject of this report; but the inestimable importance attaching to everything calculated to deprive surgery of the horrors of blood poisoning must be my excuse for having detained you so long.

1558 Wabash Avenue.

